# Supplementary material for: Resveratrol Microencapsulation into Electrosprayed Polymeric Carriers for the Treatment of Chronic, Non-Healing Wounds
Source: Pharmaceutics. 2022 Apr 13;14(4):853. doi: 10.3390/pharmaceutics14040853 (PMC9031663; doi:10.3390/pharmaceutics14040853)
Supplement: Supplementary file 1 [file pharmaceutics-14-00853-s001.zip › pharmaceutics-1662120-supplementary.pdf]

Supplementary Information

Title

Resveratrol microencapsulation into electrosprayed polymeric carriers for the treatment of chronic, non-healing wounds

Authors:

Andrea De Pieri (1), Keegan Ocorr (1), Kyle Jerreld (1), Mikkael Lamoca (1), Wolfgang Hitzl (2, 3, 4), Karin Wuertz-Kozak* (1, 5)

Affiliations:

1. Department of Biomedical Engineering, Rochester Institute of Technology (RIT), 106 Lomb Memorial Rd., Rochester, NY 14623, USA.

2 Research and Innovation Management (RIM), Biostatistics and publication of clinical trial studies, Paracelsus Medical University, 5020 Salzburg, Austria 3. Department of Ophthalmology and Optometry, Paracelsus Medical University, 5020 Salzburg, Austria

4. Research Program Experimental Ophthalmology and Glaucoma Research, Paracelsus Medical University, 5020 Salzburg, Austria

5. Schön Clinic Munich Harlaching, Spine Center, Academic Teaching Hospital and Spine Research Institute of the Paracelsus Medical University Salzburg (Austria), 81547 Munich, Germany.

*** Corresponding Authors**

Karin Wuertz-Kozak, Department of Biomedical Engineering, Rochester Institute of Technology (RIT), Rochester, NY, USA. Telephone: +1 (585)-475-7355. Email: kwbme@rit.edu

Supplementary Table S1: Primer details and gene expression assay IDs.

Gene	Gene description	Assay ID	Amplicon length
ACTA2	Actin, alpha 2, smooth muscle	Hs00426835_g1	105
ADAMTS4	ADAM metallopeptidase with thrombospondin type 1 motif 4	Hs00192708_m1	63
COL1A2	Collagen type I alpha 2 chain	Hs01028956_m1	71
CXCL8	C-X-C motif chemokine ligand 8	Hs00174103_m1	101
IL6	Interleukin 6	Hs00174131_m1	95
MMP1	Matrix metallopeptidase 1	Hs00899658_m1	64
MMP2	Matrix metallopeptidase 2	Hs01548727_m1	65
MMP3	Matrix metallopeptidase 3	Hs00968305_m1	126
MMP9	Matrix metallopeptidase 9	Hs00957562_m1	67
MMP13	Matrix metallopeptidase 13	Hs00942584_m1	91
PTGS2	Prostaglandin-endoperoxide synthase 2	Hs00153133_m1	75
TIMP1	TIMP metallopeptidase inhibitor 1	Hs01092512_g1	78
TNFAIP6	TNF alpha induced protein 6	Hs00200180_m1	60
YWHAZ	Tyrosine 3-monooxygenase/tryptophan 5-monooxygenase activation protein zeta	Hs01122445_g1	62

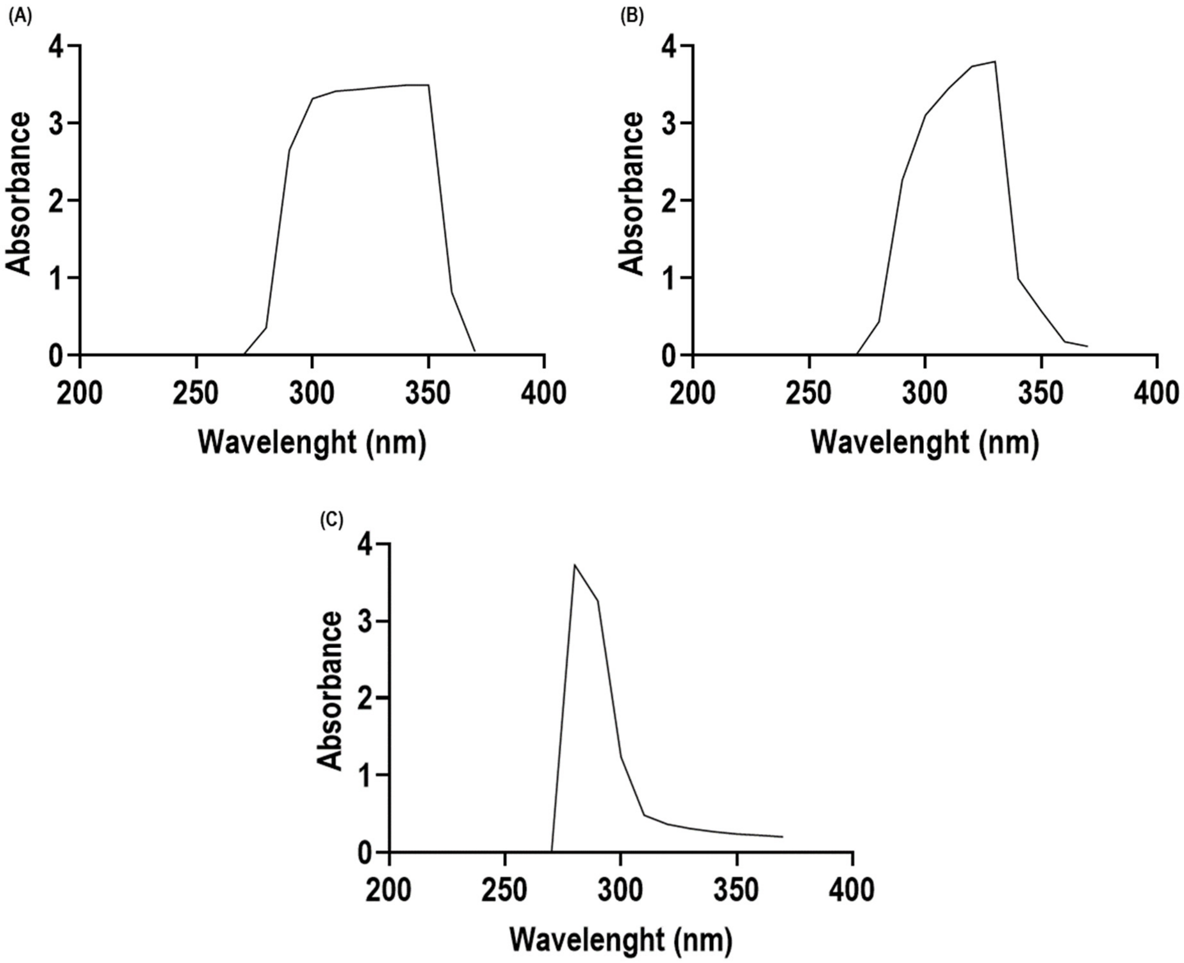

Supplementary Figure S1: The wavelength scan for a solution containing 1 mg/mL RSV dissolved in DMSO **(A)** showed a maximum absorbance wavelength of 330 nm. The wavelength scan for a solution containing 1 mg/mL 10% RSV-PCL microparticles dissolved in DMSO **(B)** showed that PCL in the solution did not affect RSV's absorbance spectrum. (**C)** The wavelength scan for a solution containing 1 mg/mL of pure PCL dissolved in DMSO.

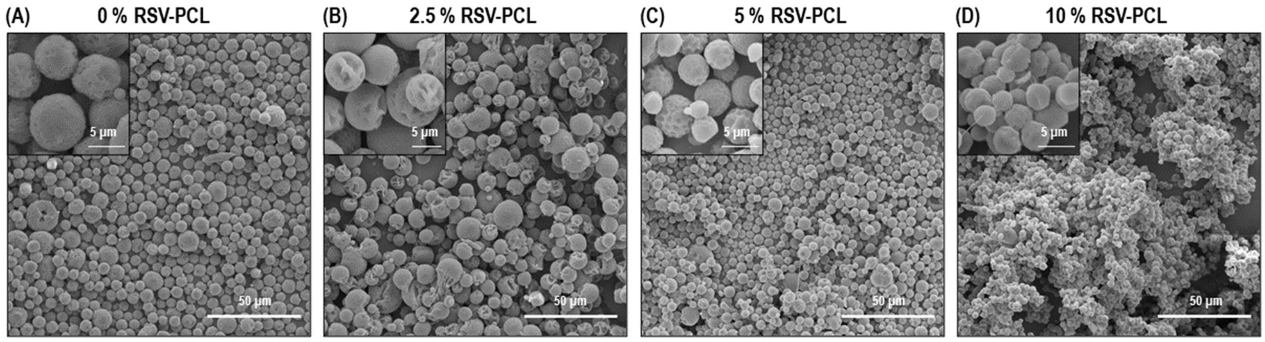

Supplementary Figure S2: Scanning electron microscopy analysis of **(A)** 0 % RSV-PCL, **(B)** 2.5 % RSV-PCL, **(C)** 5 % RSV-PCL, and **(D)** 10 % RSV-PCL revealed that all electrosprayed samples produced without PVA coating were composed of uniform (fiber-free) particles spherical in shape and exhibited a similar morphology to PVA-coated samples (n = 3 batches).

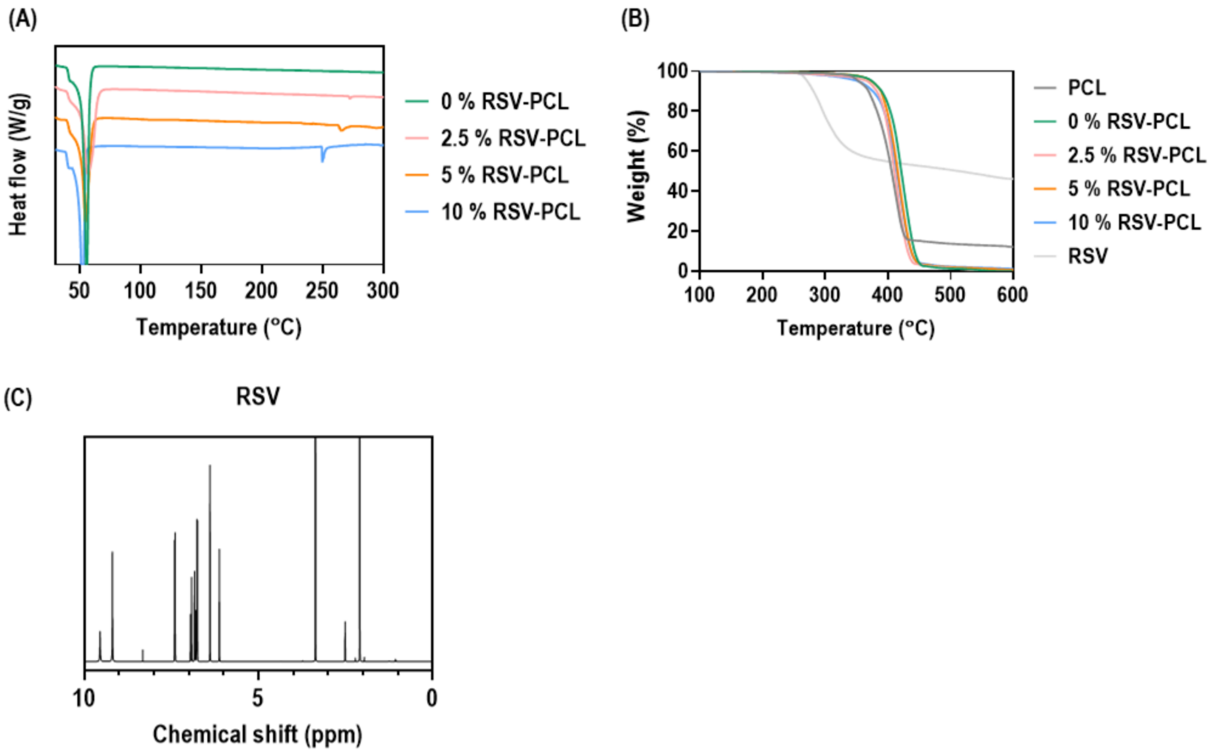

Supplementary Figure S3: DSC analysis (**A**) showed that only in rare cases, negligible amounts of crystalline RSV could be detected at 260 °C. Thermogravimetric (TGA) analysis **(B)** showed a shift of RSV weight loss upon incorporation in PCL indicating that the polymer enhanced the thermal stability of RSV (n = 3 batches). ^{1}H-NMR spectrum **(C)** of control RSV powder.

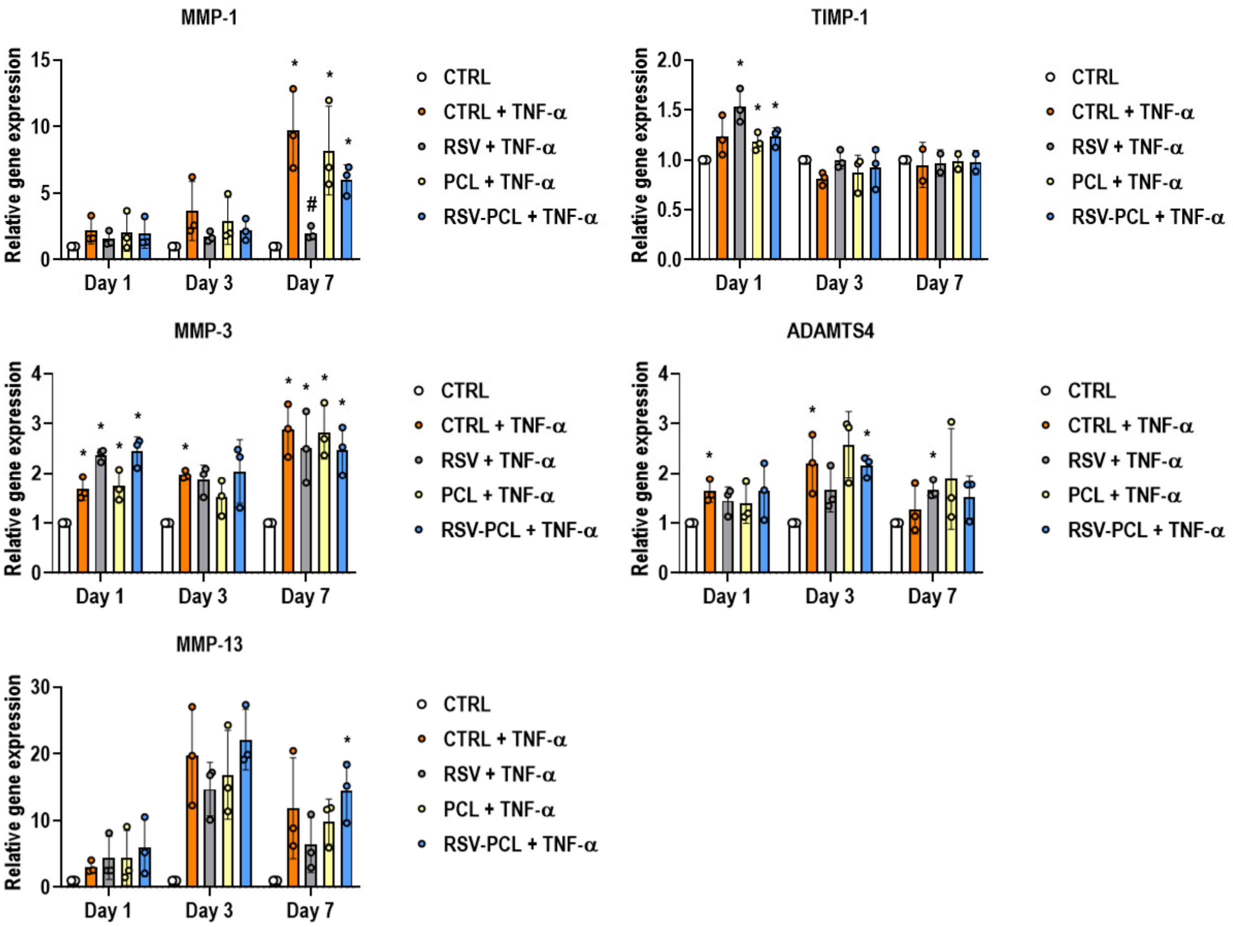

Supplementary Figure S4: Effect of 0 % RSV-PCL (PCL) and 10 % RSV-PCL (RSV-PCL) electrosprayed microparticles on gene expression of human dermal fibroblasts. RT-qPCR analysis (n = 3) revealed that RSV-PCL microparticles did not reduce ($p > 0.05$) the expression of the ECM remodeling enzymes MMP-1, MMP-3, MMP-13, TIMP-1, and ADAMTS4 in comparison to cells treated with TNF-α (50 ng/mL). Media containing 5 µM RSV was used as a positive control. The results were normalized to untreated control (CTRL) cells at each time point. * indicates statistically significant difference to CTRL group at each time point ($p < 0.05$). Individual values are displayed as dots on bar charts. Error bars represent mean ± SD. Generalized linear models and bootstrap-t tests.

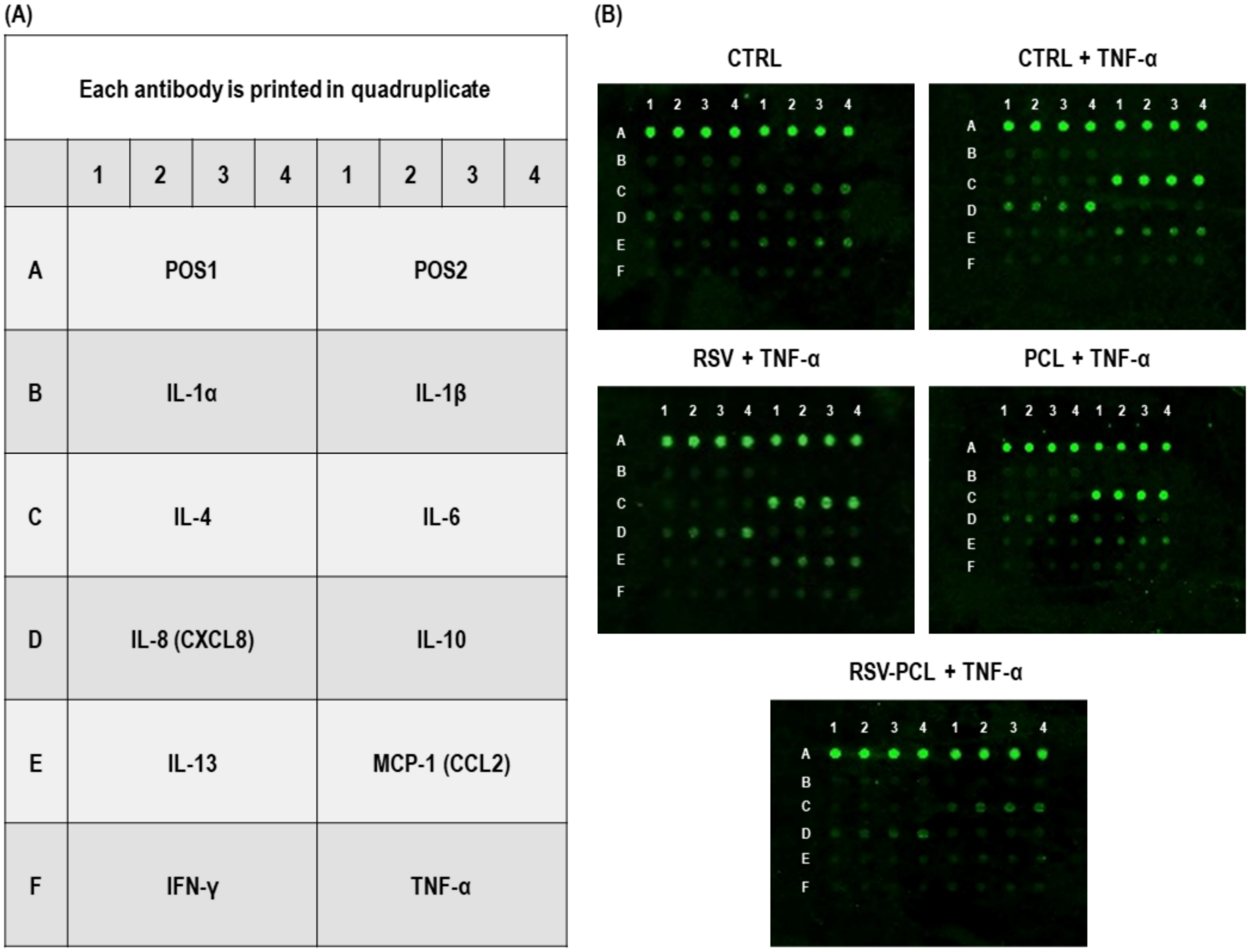

Supplementary Figure S5: Multiplex ELISA array setup **(A)** and fluorescent array scans **(B)** showed that 10 % RSV-PCL microparticles (100 µg/mL) significantly ($p < 0.05$) reduced secretion of IL-6, IL-8, and MCP-1 after 7 days of treatment.

Supplementary Table S2: Detailed statistical analysis of gene expression results.

Gene	Timepoint	Variable 1		Variable 2	Mean 1	Mean 2	Std 1	Std 2	*p*-value
IL-6	Day 1	CTRL	vs	CTRL + TNF-α	1.000	51.951	0.000	8.368	<0.001
		CTRL	vs	RSV + TNF-α	1.000	33.819	0.000	10.547	<0.001
		CTRL	vs	PCL + TNF-α	1.000	57.566	0.000	12.553	0.016
		CTRL	vs	RSV-PCL + TNF-α	1.000	22.834	0.000	3.576	<0.001
		CTRL + TNF-α	vs	RSV + TNF-α	51.951	33.819	8.368	10.547	0.250
		CTRL + TNF-α	vs	PCL + TNF-α	51.951	57.566	8.368	12.553	0.246
		CTRL + TNF-α	vs	RSV-PCL + TNF-α	51.951	22.834	8.368	3.576	0.025
		RSV + TNF-α	vs	PCL + TNF-α	33.819	57.566	10.547	12.553	0.123
		RSV + TNF-α	vs	RSV-PCL + TNF-α	33.819	22.834	10.547	3.576	0.250
		PCL + TNF-α	vs	RSV-PCL + TNF-α	57.566	22.834	12.553	3.576	<0.001
	Day 3	CTRL	vs	CTRL + TNF-α	1.000	18.166	0.000	2.723	<0.001
		CTRL	vs	RSV + TNF-α	1.000	11.387	0.000	1.095	<0.001
		CTRL	vs	PCL + TNF-α	1.000	15.946	0.000	1.712	<0.001
		CTRL	vs	RSV-PCL + TNF-α	1.000	8.080	0.000	1.248	<0.001
		CTRL + TNF-α	vs	RSV + TNF-α	18.166	11.387	2.723	1.095	<0.001
		CTRL + TNF-α	vs	PCL + TNF-α	18.166	15.946	2.723	1.712	0.248
		CTRL + TNF-α	vs	RSV-PCL + TNF-α	18.166	8.080	2.723	1.248	<0.001
		RSV + TNF-α	vs	PCL + TNF-α	11.387	15.946	1.095	1.712	<0.001
		RSV + TNF-α	vs	RSV-PCL + TNF-α	11.387	8.080	1.095	1.248	<0.001
		PCL + TNF-α	vs	RSV-PCL + TNF-α	15.946	8.080	1.712	1.248	<0.001
	Day 7	CTRL	vs	CTRL + TNF-α	1.000	17.193	0.000	1.065	<0.001
		CTRL	vs	RSV + TNF-α	1.000	9.812	0.000	1.399	0.008
		CTRL	vs	PCL + TNF-α	1.000	16.399	0.000	3.954	<0.001
		CTRL	vs	RSV-PCL + TNF-α	1.000	10.028	0.000	1.569	<0.001
		CTRL + TNF-α	vs	RSV + TNF-α	17.193	9.812	1.065	1.399	<0.001
		CTRL + TNF-α	vs	PCL + TNF-α	17.193	16.399	1.065	3.954	0.485

		CTRL + TNF-α	vs	RSV-PCL + TNF-α	17.193	10.028	1.065	1.569	0.034
		RSV + TNF-α	vs	PCL + TNF-α	9.812	16.399	1.399	3.954	0.237
		RSV + TNF-α	vs	RSV-PCL + TNF-α	9.812	10.028	1.399	1.569	0.739
		PCL + TNF-α	vs	RSV-PCL + TNF-α	16.399	10.028	3.954	1.569	<0.001
IL-8	Day 1	CTRL	vs	CTRL + TNF-α	1.000	256.218	0.000	28.441	<0.001
		CTRL	vs	RSV + TNF-α	1.000	142.197	0.000	35.033	0.019
		CTRL	vs	PCL + TNF-α	1.000	235.040	0.000	34.260	<0.001
		CTRL	vs	RSV-PCL + TNF-α	1.000	131.899	0.000	29.374	0.016
		CTRL + TNF-α	vs	RSV + TNF-α	256.218	142.197	28.441	35.033	<0.001
		CTRL + TNF-α	vs	PCL + TNF-α	256.218	235.040	28.441	34.260	0.246
		CTRL + TNF-α	vs	RSV-PCL + TNF-α	256.218	131.899	28.441	29.374	0.010
		RSV + TNF-α	vs	PCL + TNF-α	142.197	235.040	35.033	34.260	<0.001
		RSV + TNF-α	vs	RSV-PCL + TNF-α	142.197	131.899	35.033	29.374	<0.001
		PCL + TNF-α	vs	RSV-PCL + TNF-α	235.040	131.899	34.260	29.374	0.002
	Day 3	CTRL	vs	CTRL + TNF-α	1.000	817.277	0.000	176.229	0.015
		CTRL	vs	RSV + TNF-α	1.000	264.461	0.000	89.612	0.036
		CTRL	vs	PCL + TNF-α	1.000	762.407	0.000	88.952	<0.001
		CTRL	vs	RSV-PCL + TNF-α	1.000	221.927	0.000	40.626	<0.001
		CTRL + TNF-α	vs	RSV + TNF-α	817.277	264.461	176.229	89.612	<0.001
		CTRL + TNF-α	vs	PCL + TNF-α	817.277	762.407	176.229	88.952	0.359
		CTRL + TNF-α	vs	RSV-PCL + TNF-α	817.277	221.927	176.229	40.626	0.041
		RSV + TNF-α	vs	PCL + TNF-α	264.461	762.407	89.612	88.952	<0.001
		RSV + TNF-α	vs	RSV-PCL + TNF-α	264.461	221.927	89.612	40.626	0.380
		PCL + TNF-α	vs	RSV-PCL + TNF-α	762.407	221.927	88.952	40.626	<0.001
	Day 7	CTRL	vs	CTRL + TNF-α	1.000	2144.710	0.000	761.985	<0.001
		CTRL	vs	RSV + TNF-α	1.000	484.774	0.000	138.568	<0.001
		CTRL	vs	PCL + TNF-α	1.000	1851.520	0.000	375.316	<0.001
		CTRL	vs	RSV-PCL + TNF-α	1.000	532.622	0.000	118.948	<0.001

		CTRL + TNF-α	vs	RSV + TNF-α	2144.710	484.774	761.985	138.568	<0.001
		CTRL + TNF-α	vs	PCL + TNF-α	2144.710	1851.520	761.985	375.316	0.370
		CTRL + TNF-α	vs	RSV-PCL + TNF-α	2144.710	532.622	761.985	118.948	<0.001
		RSV + TNF-α	vs	PCL + TNF-α	484.774	1851.520	138.568	375.316	<0.001
		RSV + TNF-α	vs	RSV-PCL + TNF-α	484.774	532.622	138.568	118.948	0.250
		PCL + TNF-α	vs	RSV-PCL + TNF-α	1851.520	532.622	375.316	118.948	<0.001
COX-2	Day 1	CTRL	vs	CTRL + TNF-α	1.000	12.747	0.000	3.330	<0.001
		CTRL	vs	RSV + TNF-α	1.000	3.839	0.000	0.610	<0.001
		CTRL	vs	PCL + TNF-α	1.000	11.401	0.000	2.843	<0.001
		CTRL	vs	RSV-PCL + TNF-α	1.000	2.232	0.000	0.558	<0.001
		CTRL + TNF-α	vs	RSV + TNF-α	12.747	3.839	3.330	0.610	<0.001
		CTRL + TNF-α	vs	PCL + TNF-α	12.747	11.401	3.330	2.843	0.497
		CTRL + TNF-α	vs	RSV-PCL + TNF-α	12.747	2.232	3.330	0.558	<0.001
		RSV + TNF-α	vs	PCL + TNF-α	3.839	11.401	0.610	2.843	0.120
		RSV + TNF-α	vs	RSV-PCL + TNF-α	3.839	2.232	0.610	0.558	0.254
		PCL + TNF-α	vs	RSV-PCL + TNF-α	11.401	2.232	2.843	0.558	<0.001
	Day 3	CTRL	vs	CTRL + TNF-α	1.000	7.475	0.000	1.871	<0.001
		CTRL	vs	RSV + TNF-α	1.000	1.365	0.000	0.548	0.377
		CTRL	vs	PCL + TNF-α	1.000	4.372	0.000	0.608	<0.001
		CTRL	vs	RSV-PCL + TNF-α	1.000	1.375	0.000	0.296	0.259
		CTRL + TNF-α	vs	RSV + TNF-α	7.475	1.365	1.871	0.548	0.047
		CTRL + TNF-α	vs	PCL + TNF-α	7.475	4.372	1.871	0.608	<0.001
		CTRL + TNF-α	vs	RSV-PCL + TNF-α	7.475	1.375	1.871	0.296	0.023
		RSV + TNF-α	vs	PCL + TNF-α	1.365	4.372	0.548	0.608	<0.001
		RSV + TNF-α	vs	RSV-PCL + TNF-α	1.365	1.375	0.548	0.296	0.748
		PCL + TNF-α	vs	RSV-PCL + TNF-α	4.372	1.375	0.608	0.296	<0.001
	Day 7	CTRL	vs	CTRL + TNF-α	1.000	7.101	0.000	1.438	<0.001
		CTRL	vs	RSV + TNF-α	1.000	1.255	0.000	0.204	0.239

		CTRL	vs	PCL + TNF-α	1.000	6.622	0.000	1.144	<0.001
		CTRL	vs	RSV-PCL + TNF-α	1.000	2.297	0.000	0.768	<0.001
		CTRL + TNF-α	vs	RSV + TNF-α	7.101	1.255	1.438	0.204	<0.001
		CTRL + TNF-α	vs	PCL + TNF-α	7.101	6.622	1.438	1.144	0.495
		CTRL + TNF-α	vs	RSV-PCL + TNF-α	7.101	2.297	1.438	0.768	<0.001
		RSV + TNF-α	vs	PCL + TNF-α	1.255	6.622	0.204	1.144	<0.001
		RSV + TNF-α	vs	RSV-PCL + TNF-α	1.255	2.297	0.204	0.768	0.248
		PCL + TNF-α	vs	RSV-PCL + TNF-α	6.622	2.297	1.144	0.768	<0.001
MMP-1	Day 1	CTRL	vs	CTRL + TNF-α	1.000	2.178	0.000	0.990	0.249
		CTRL	vs	RSV + TNF-α	1.000	1.565	0.000	0.551	0.248
		CTRL	vs	PCL + TNF-α	1.000	2.083	0.000	1.431	0.382
		CTRL	vs	RSV-PCL + TNF-α	1.000	1.970	0.000	1.111	0.256
		CTRL + TNF-α	vs	RSV + TNF-α	2.178	1.565	0.990	0.551	0.256
		CTRL + TNF-α	vs	PCL + TNF-α	2.178	2.083	0.990	1.431	0.617
		CTRL + TNF-α	vs	RSV-PCL + TNF-α	2.178	1.970	0.990	1.111	0.117
		RSV + TNF-α	vs	PCL + TNF-α	1.565	2.083	0.551	1.431	0.374
		RSV + TNF-α	vs	RSV-PCL + TNF-α	1.565	1.970	0.551	1.111	0.245
		PCL + TNF-α	vs	RSV-PCL + TNF-α	2.083	1.970	1.431	1.111	0.510
	Day 3	CTRL	vs	CTRL + TNF-α	1.000	3.666	0.000	2.221	0.252
		CTRL	vs	RSV + TNF-α	1.000	1.727	0.000	0.382	0.080
		CTRL	vs	PCL + TNF-α	1.000	2.922	0.000	1.766	0.255
		CTRL	vs	RSV-PCL + TNF-α	1.000	2.233	0.000	0.811	0.119
		CTRL + TNF-α	vs	RSV + TNF-α	3.666	1.727	2.221	0.382	0.241
		CTRL + TNF-α	vs	PCL + TNF-α	3.666	2.922	2.221	1.766	0.238
		CTRL + TNF-α	vs	RSV-PCL + TNF-α	3.666	2.233	2.221	0.811	0.247
		RSV + TNF-α	vs	PCL + TNF-α	1.727	2.922	0.382	1.766	0.241
		RSV + TNF-α	vs	RSV-PCL + TNF-α	1.727	2.233	0.382	0.811	0.127
		PCL + TNF-α	vs	RSV-PCL + TNF-α	2.922	2.233	1.766	0.811	0.369

	Day 7	CTRL	vs	CTRL + TNF-α	1.000	9.694	0.000	2.992	<0.001
		CTRL	vs	RSV + TNF-α	1.000	2.000	0.000	0.477	0.248
		CTRL	vs	PCL + TNF-α	1.000	8.200	0.000	3.334	0.035
		CTRL	vs	RSV-PCL + TNF-α	1.000	6.035	0.000	1.111	<0.001
		CTRL + TNF-α	vs	RSV + TNF-α	9.694	2.000	2.992	0.477	<0.001
		CTRL + TNF-α	vs	PCL + TNF-α	9.694	8.200	2.992	3.334	0.508
		CTRL + TNF-α	vs	RSV-PCL + TNF-α	9.694	6.035	2.992	1.111	0.255
		RSV + TNF-α	vs	PCL + TNF-α	2.000	8.200	0.477	3.334	0.117
		RSV + TNF-α	vs	RSV-PCL + TNF-α	2.000	6.035	0.477	1.111	<0.001
		PCL + TNF-α	vs	RSV-PCL + TNF-α	8.200	6.035	3.334	1.111	0.251
MMP-2	Day 1	CTRL	vs	CTRL + TNF-α	1.000	2.448	0.000	0.527	<0.001
		CTRL	vs	RSV + TNF-α	1.000	1.663	0.000	0.646	0.243
		CTRL	vs	PCL + TNF-α	1.000	1.840	0.000	0.705	0.129
		CTRL	vs	RSV-PCL + TNF-α	1.000	1.457	0.000	0.745	0.236
		CTRL + TNF-α	vs	RSV + TNF-α	2.448	1.663	0.527	0.646	0.046
		CTRL + TNF-α	vs	PCL + TNF-α	2.448	1.840	0.527	0.705	0.251
		CTRL + TNF-α	vs	RSV-PCL + TNF-α	2.448	1.457	0.527	0.745	<0.001
		RSV + TNF-α	vs	PCL + TNF-α	1.663	1.840	0.646	0.705	0.365
		RSV + TNF-α	vs	RSV-PCL + TNF-α	1.663	1.457	0.646	0.745	<0.001
		PCL + TNF-α	vs	RSV-PCL + TNF-α	1.840	1.457	0.705	0.745	0.250
	Day 3	CTRL	vs	CTRL + TNF-α	1.000	1.437	0.000	0.232	<0.001
		CTRL	vs	RSV + TNF-α	1.000	0.968	0.000	0.361	0.488
		CTRL	vs	PCL + TNF-α	1.000	1.255	0.000	0.380	0.243
		CTRL	vs	RSV-PCL + TNF-α	1.000	0.829	0.000	0.451	0.503
		CTRL + TNF-α	vs	RSV + TNF-α	1.437	0.968	0.232	0.361	<0.001
		CTRL + TNF-α	vs	PCL + TNF-α	1.437	1.255	0.232	0.380	0.252
		CTRL + TNF-α	vs	RSV-PCL + TNF-α	1.437	0.829	0.232	0.451	<0.001
		RSV + TNF-α	vs	PCL + TNF-α	0.968	1.255	0.361	0.380	0.125

		RSV + TNF-α	vs	RSV-PCL + TNF-α	0.968	0.829	0.361	0.451	0.121
		PCL + TNF-α	vs	RSV-PCL + TNF-α	1.255	0.829	0.380	0.451	0.243
	Day 7	CTRL	vs	CTRL + TNF-α	1.000	1.438	0.000	0.577	0.249
		CTRL	vs	RSV + TNF-α	1.000	1.254	0.000	0.278	0.248
		CTRL	vs	PCL + TNF-α	1.000	1.659	0.000	0.332	<0.001
		CTRL	vs	RSV-PCL + TNF-α	1.000	1.294	0.000	0.492	0.371
		CTRL + TNF-α	vs	RSV + TNF-α	1.438	1.254	0.577	0.278	0.384
		CTRL + TNF-α	vs	PCL + TNF-α	1.438	1.659	0.577	0.332	0.382
		CTRL + TNF-α	vs	RSV-PCL + TNF-α	1.438	1.294	0.577	0.492	0.504
		RSV + TNF-α	vs	PCL + TNF-α	1.254	1.659	0.278	0.332	<0.001
		RSV + TNF-α	vs	RSV-PCL + TNF-α	1.254	1.294	0.278	0.492	0.511
		PCL + TNF-α	vs	RSV-PCL + TNF-α	1.659	1.294	0.332	0.492	<0.001
MMP-3	Day 1	CTRL	vs	CTRL + TNF-α	1.000	1.678	0.000	0.222	<0.001
		CTRL	vs	RSV + TNF-α	1.000	2.367	0.000	0.124	<0.001
		CTRL	vs	PCL + TNF-α	1.000	1.738	0.000	0.308	<0.001
		CTRL	vs	RSV-PCL + TNF-α	1.000	2.443	0.000	0.292	<0.001
		CTRL + TNF-α	vs	RSV + TNF-α	1.678	2.367	0.222	0.124	0.250
		CTRL + TNF-α	vs	PCL + TNF-α	1.678	1.738	0.222	0.308	0.754
		CTRL + TNF-α	vs	RSV-PCL + TNF-α	1.678	2.443	0.222	0.292	0.245
		RSV + TNF-α	vs	PCL + TNF-α	2.367	1.738	0.124	0.308	0.255
		RSV + TNF-α	vs	RSV-PCL + TNF-α	2.367	2.443	0.124	0.292	0.388
		PCL + TNF-α	vs	RSV-PCL + TNF-α	1.738	2.443	0.308	0.292	<0.001
	Day 3	CTRL	vs	CTRL + TNF-α	1.000	1.967	0.000	0.080	<0.001
		CTRL	vs	RSV + TNF-α	1.000	1.862	0.000	0.306	0.133
		CTRL	vs	PCL + TNF-α	1.000	1.510	0.000	0.356	<0.001
		CTRL	vs	RSV-PCL + TNF-α	1.000	2.038	0.000	0.640	0.259
		CTRL + TNF-α	vs	RSV + TNF-α	1.967	1.862	0.080	0.306	0.506
		CTRL + TNF-α	vs	PCL + TNF-α	1.967	1.510	0.080	0.356	0.241

		CTRL + TNF-α	vs	RSV-PCL + TNF-α	1.967	2.038	0.080	0.640	0.753
		RSV + TNF-α	vs	PCL + TNF-α	1.862	1.510	0.306	0.356	<0.001
		RSV + TNF-α	vs	RSV-PCL + TNF-α	1.862	2.038	0.306	0.640	0.488
		PCL + TNF-α	vs	RSV-PCL + TNF-α	1.510	2.038	0.356	0.640	0.122
	Day 7	CTRL	vs	CTRL + TNF-α	1.000	2.871	0.000	0.529	<0.001
		CTRL	vs	RSV + TNF-α	1.000	2.517	0.000	0.718	<0.001
		CTRL	vs	PCL + TNF-α	1.000	2.826	0.000	0.539	<0.001
		CTRL	vs	RSV-PCL + TNF-α	1.000	2.470	0.000	0.484	<0.001
		CTRL + TNF-α	vs	RSV + TNF-α	2.871	2.517	0.529	0.718	0.367
		CTRL + TNF-α	vs	PCL + TNF-α	2.871	2.826	0.529	0.539	0.494
		CTRL + TNF-α	vs	RSV-PCL + TNF-α	2.871	2.470	0.529	0.484	0.241
		RSV + TNF-α	vs	PCL + TNF-α	2.517	2.826	0.718	0.539	0.500
		RSV + TNF-α	vs	RSV-PCL + TNF-α	2.517	2.470	0.718	0.484	0.751
		PCL + TNF-α	vs	RSV-PCL + TNF-α	2.826	2.470	0.539	0.484	<0.001
MMP-9	Day 1	CTRL	vs	CTRL + TNF-α	1.000	43.767	0.000	12.959	0.029
		CTRL	vs	RSV + TNF-α	1.000	13.082	0.000	5.108	0.123
		CTRL	vs	PCL + TNF-α	1.000	22.485	0.000	6.570	<0.001
		CTRL	vs	RSV-PCL + TNF-α	1.000	13.661	0.000	3.790	<0.001
		CTRL + TNF-α	vs	RSV + TNF-α	43.767	13.082	12.959	5.108	<0.001
		CTRL + TNF-α	vs	PCL + TNF-α	43.767	22.485	12.959	6.570	<0.001
		CTRL + TNF-α	vs	RSV-PCL + TNF-α	43.767	13.661	12.959	3.790	0.031
		RSV + TNF-α	vs	PCL + TNF-α	13.082	22.485	5.108	6.570	<0.001
		RSV + TNF-α	vs	RSV-PCL + TNF-α	13.082	13.661	5.108	3.790	0.492
		PCL + TNF-α	vs	RSV-PCL + TNF-α	22.485	13.661	6.570	3.790	<0.001
	Day 3	CTRL	vs	CTRL + TNF-α	1.000	41.279	0.000	13.601	<0.001
		CTRL	vs	RSV + TNF-α	1.000	24.285	0.000	9.519	0.245
		CTRL	vs	PCL + TNF-α	1.000	27.305	0.000	8.600	<0.001
		CTRL	vs	RSV-PCL + TNF-α	1.000	22.608	0.000	5.692	<0.001

		CTRL + TNF-α	vs	RSV + TNF-α	41.279	24.285	13.601	9.519	<0.001
		CTRL + TNF-α	vs	PCL + TNF-α	41.279	27.305	13.601	8.600	<0.001
		CTRL + TNF-α	vs	RSV-PCL + TNF-α	41.279	22.608	13.601	5.692	0.245
		RSV + TNF-α	vs	PCL + TNF-α	24.285	27.305	9.519	8.600	0.252
		RSV + TNF-α	vs	RSV-PCL + TNF-α	24.285	22.608	9.519	5.692	0.511
		PCL + TNF-α	vs	RSV-PCL + TNF-α	27.305	22.608	8.600	5.692	<0.001
	Day 7	CTRL	vs	CTRL + TNF-α	1.000	54.991	0.000	7.624	0.006
		CTRL	vs	RSV + TNF-α	1.000	27.840	0.000	9.295	<0.001
		CTRL	vs	PCL + TNF-α	1.000	31.546	0.000	3.952	<0.001
		CTRL	vs	RSV-PCL + TNF-α	1.000	28.054	0.000	5.565	<0.001
		CTRL + TNF-α	vs	RSV + TNF-α	54.991	27.840	7.624	9.295	<0.001
		CTRL + TNF-α	vs	PCL + TNF-α	54.991	31.546	7.624	3.952	<0.001
		CTRL + TNF-α	vs	RSV-PCL + TNF-α	54.991	28.054	7.624	5.565	<0.001
		RSV + TNF-α	vs	PCL + TNF-α	27.840	31.546	9.295	3.952	0.502
		RSV + TNF-α	vs	RSV-PCL + TNF-α	27.840	28.054	9.295	5.565	0.752
		PCL + TNF-α	vs	RSV-PCL + TNF-α	31.546	28.054	3.952	5.565	0.375
MMP-13	Day 1	CTRL	vs	CTRL + TNF-α	1.000	3.034	0.000	0.887	0.244
		CTRL	vs	RSV + TNF-α	1.000	4.404	0.000	3.239	0.249
		CTRL	vs	PCL + TNF-α	1.000	4.374	0.000	4.107	0.254
		CTRL	vs	RSV-PCL + TNF-α	1.000	5.944	0.000	4.271	0.122
		CTRL + TNF-α	vs	RSV + TNF-α	3.034	4.404	0.887	3.239	0.363
		CTRL + TNF-α	vs	PCL + TNF-α	3.034	4.374	0.887	4.107	0.484
		CTRL + TNF-α	vs	RSV-PCL + TNF-α	3.034	5.944	0.887	4.271	0.238
		RSV + TNF-α	vs	PCL + TNF-α	4.404	4.374	3.239	4.107	0.746
		RSV + TNF-α	vs	RSV-PCL + TNF-α	4.404	5.944	3.239	4.271	0.241
		PCL + TNF-α	vs	RSV-PCL + TNF-α	4.374	5.944	4.107	4.271	<0.001
	Day 3	CTRL	vs	CTRL + TNF-α	1.000	19.694	0.000	7.395	<0.001
		CTRL	vs	RSV + TNF-α	1.000	14.716	0.000	3.996	0.027

		CTRL	vs	PCL + TNF-α	1.000	16.871	0.000	6.671	<0.001
		CTRL	vs	RSV-PCL + TNF-α	1.000	22.144	0.000	4.543	0.015
		CTRL + TNF-α	vs	RSV + TNF-α	19.694	14.716	7.395	3.996	0.247
		CTRL + TNF-α	vs	PCL + TNF-α	19.694	16.871	7.395	6.671	<0.001
		CTRL + TNF-α	vs	RSV-PCL + TNF-α	19.694	22.144	7.395	4.543	0.499
		RSV + TNF-α	vs	PCL + TNF-α	14.716	16.871	3.996	6.671	0.372
		RSV + TNF-α	vs	RSV-PCL + TNF-α	14.716	22.144	3.996	4.543	0.246
		PCL + TNF-α	vs	RSV-PCL + TNF-α	16.871	22.144	6.671	4.543	0.373
	Day 7	CTRL	vs	CTRL + TNF-α	1.000	11.842	0.000	7.586	0.250
		CTRL	vs	RSV + TNF-α	1.000	6.369	0.000	4.126	0.248
		CTRL	vs	PCL + TNF-α	1.000	9.835	0.000	3.373	0.251
		CTRL	vs	RSV-PCL + TNF-α	1.000	14.401	0.000	4.423	<0.001
		CTRL + TNF-α	vs	RSV + TNF-α	11.842	6.369	7.586	4.126	0.261
		CTRL + TNF-α	vs	PCL + TNF-α	11.842	9.835	7.586	3.373	0.492
		CTRL + TNF-α	vs	RSV-PCL + TNF-α	11.842	14.401	7.586	4.423	0.377
		RSV + TNF-α	vs	PCL + TNF-α	6.369	9.835	4.126	3.373	0.127
		RSV + TNF-α	vs	RSV-PCL + TNF-α	6.369	14.401	4.126	4.423	<0.001
		PCL + TNF-α	vs	RSV-PCL + TNF-α	9.835	14.401	3.373	4.423	0.261
TIMP-1	Day 1	CTRL	vs	CTRL + TNF-α	1.000	1.235	0.000	0.201	0.118
		CTRL	vs	RSV + TNF-α	1.000	1.533	0.000	0.169	0.031
		CTRL	vs	PCL + TNF-α	1.000	1.180	0.000	0.091	0.075
		CTRL	vs	RSV-PCL + TNF-α	1.000	1.229	0.000	0.091	0.048
		CTRL + TNF-α	vs	RSV + TNF-α	1.235	1.533	0.201	0.169	0.125
		CTRL + TNF-α	vs	PCL + TNF-α	1.235	1.180	0.201	0.091	0.502
		CTRL + TNF-α	vs	RSV-PCL + TNF-α	1.235	1.229	0.201	0.091	0.737
		RSV + TNF-α	vs	PCL + TNF-α	1.533	1.180	0.169	0.091	<0.001
		RSV + TNF-α	vs	RSV-PCL + TNF-α	1.533	1.229	0.169	0.091	0.248
		PCL + TNF-α	vs	RSV-PCL + TNF-α	1.180	1.229	0.091	0.091	0.490

	Day 3	CTRL	vs	CTRL + TNF-α	1.000	0.813	0.000	0.064	<0.001
		CTRL	vs	RSV + TNF-α	1.000	1.000	0.000	0.091	0.746
		CTRL	vs	PCL + TNF-α	1.000	0.871	0.000	0.176	0.258
		CTRL	vs	RSV-PCL + TNF-α	1.000	0.924	0.000	0.202	0.378
		CTRL + TNF-α	vs	RSV + TNF-α	0.813	1.000	0.064	0.091	0.256
		CTRL + TNF-α	vs	PCL + TNF-α	0.813	0.871	0.064	0.176	0.367
		CTRL + TNF-α	vs	RSV-PCL + TNF-α	0.813	0.924	0.064	0.202	0.492
		RSV + TNF-α	vs	PCL + TNF-α	1.000	0.871	0.091	0.176	0.498
		RSV + TNF-α	vs	RSV-PCL + TNF-α	1.000	0.924	0.091	0.202	0.499
		PCL + TNF-α	vs	RSV-PCL + TNF-α	0.871	0.924	0.176	0.202	0.629
	Day 7	CTRL	vs	CTRL + TNF-α	1.000	0.950	0.000	0.226	0.804
		CTRL	vs	RSV + TNF-α	1.000	0.969	0.000	0.132	0.793
		CTRL	vs	PCL + TNF-α	1.000	0.984	0.000	0.107	0.863
		CTRL	vs	RSV-PCL + TNF-α	1.000	0.974	0.000	0.121	0.808
		CTRL + TNF-α	vs	RSV + TNF-α	0.950	0.969	0.226	0.132	0.821
		CTRL + TNF-α	vs	PCL + TNF-α	0.950	0.984	0.226	0.107	0.755
		CTRL + TNF-α	vs	RSV-PCL + TNF-α	0.950	0.974	0.226	0.121	0.800
		RSV + TNF-α	vs	PCL + TNF-α	0.969	0.984	0.132	0.107	0.557
		RSV + TNF-α	vs	RSV-PCL + TNF-α	0.969	0.974	0.132	0.121	0.644
		PCL + TNF-α	vs	RSV-PCL + TNF-α	0.984	0.974	0.107	0.121	0.500
Col 1	Day 1	CTRL	vs	CTRL + TNF-α	1.000	1.071	0.000	0.325	0.741
		CTRL	vs	RSV + TNF-α	1.000	1.095	0.000	0.507	0.776
		CTRL	vs	PCL + TNF-α	1.000	0.735	0.000	0.331	0.299
		CTRL	vs	RSV-PCL + TNF-α	1.000	0.907	0.000	0.187	0.479
		CTRL + TNF-α	vs	RSV + TNF-α	1.071	1.095	0.325	0.507	0.841
		CTRL + TNF-α	vs	PCL + TNF-α	1.071	0.735	0.325	0.331	0.396
		CTRL + TNF-α	vs	RSV-PCL + TNF-α	1.071	0.907	0.325	0.187	0.360
		RSV + TNF-α	vs	PCL + TNF-α	1.095	0.735	0.507	0.331	0.473

		RSV + TNF-α	vs	RSV-PCL + TNF-α	1.095	0.907	0.507	0.187	0.511
		PCL + TNF-α	vs	RSV-PCL + TNF-α	0.735	0.907	0.331	0.187	0.428
	Day 3	CTRL	vs	CTRL + TNF-α	1.000	0.756	0.000	0.172	0.133
		CTRL	vs	RSV + TNF-α	1.000	0.642	0.000	0.071	0.0129
		CTRL	vs	PCL + TNF-α	1.000	0.750	0.000	0.272	0.252
		CTRL	vs	RSV-PCL + TNF-α	1.000	0.710	0.000	0.264	0.196
		CTRL + TNF-α	vs	RSV + TNF-α	0.756	0.642	0.172	0.071	0.442
		CTRL + TNF-α	vs	PCL + TNF-α	0.756	0.750	0.172	0.272	0.942
		CTRL + TNF-α	vs	RSV-PCL + TNF-α	0.756	0.710	0.172	0.264	0.476
		RSV + TNF-α	vs	PCL + TNF-α	0.642	0.750	0.071	0.272	0.573
		RSV + TNF-α	vs	RSV-PCL + TNF-α	0.642	0.710	0.071	0.264	0.727
		PCL + TNF-α	vs	RSV-PCL + TNF-α	0.750	0.710	0.272	0.264	0.526
	Day 7	CTRL	vs	CTRL + TNF-α	1.000	0.455	0.000	0.252	0.064
		CTRL	vs	RSV + TNF-α	1.000	0.472	0.000	0.119	0.016
		CTRL	vs	PCL + TNF-α	1.000	0.615	0.000	0.245	0.112
		CTRL	vs	RSV-PCL + TNF-α	1.000	0.458	0.000	0.252	0.064
		CTRL + TNF-α	vs	RSV + TNF-α	0.455	0.472	0.252	0.119	0.867
		CTRL + TNF-α	vs	PCL + TNF-α	0.455	0.615	0.252	0.245	0.391
		CTRL + TNF-α	vs	RSV-PCL + TNF-α	0.455	0.458	0.252	0.252	0.980
		RSV + TNF-α	vs	PCL + TNF-α	0.472	0.615	0.119	0.245	0.462
		RSV + TNF-α	vs	RSV-PCL + TNF-α	0.472	0.458	0.119	0.252	0.932
		PCL + TNF-α	vs	RSV-PCL + TNF-α	0.615	0.458	0.245	0.252	0.047
α-SMA	Day 1	CTRL	vs	CTRL + TNF-α	1.000	0.790	0.000	0.130	0.108
		CTRL	vs	RSV + TNF-α	1.000	0.941	0.000	0.255	0.725
		CTRL	vs	PCL + TNF-α	1.000	0.752	0.000	0.169	0.126
		CTRL	vs	RSV-PCL + TNF-α	1.000	1.206	0.000	0.101	0.071
		CTRL + TNF-α	vs	RSV + TNF-α	0.790	0.941	0.130	0.255	0.216
		CTRL + TNF-α	vs	PCL + TNF-α	0.790	0.752	0.130	0.169	0.449

		CTRL + TNF-α	vs	RSV-PCL + TNF-α	0.790	1.206	0.130	0.101	0.011
		RSV + TNF-α	vs	PCL + TNF-α	0.941	0.752	0.255	0.169	0.223
		RSV + TNF-α	vs	RSV-PCL + TNF-α	0.941	1.206	0.255	0.101	0.177
		PCL + TNF-α	vs	RSV-PCL + TNF-α	0.752	1.206	0.169	0.101	0.008
	Day 3	CTRL	vs	CTRL + TNF-α	1.000	1.597	0.000	0.124	<0.001
		CTRL	vs	RSV + TNF-α	1.000	0.523	0.000	0.048	<0.001
		CTRL	vs	PCL + TNF-α	1.000	1.216	0.000	0.070	0.033
		CTRL	vs	RSV-PCL + TNF-α	1.000	0.602	0.000	0.057	<0.001
		CTRL + TNF-α	vs	RSV + TNF-α	1.597	0.523	0.124	0.048	<0.001
		CTRL + TNF-α	vs	PCL + TNF-α	1.597	1.216	0.124	0.070	<0.001
		CTRL + TNF-α	vs	RSV-PCL + TNF-α	1.597	0.602	0.124	0.057	<0.001
		RSV + TNF-α	vs	PCL + TNF-α	0.523	1.216	0.048	0.070	<0.001
		RSV + TNF-α	vs	RSV-PCL + TNF-α	0.523	0.602	0.048	0.057	<0.001
		PCL + TNF-α	vs	RSV-PCL + TNF-α	1.216	0.602	0.070	0.057	<0.001
	Day 7	CTRL	vs	CTRL + TNF-α	1.000	1.717	0.000	0.549	0.152
		CTRL	vs	RSV + TNF-α	1.000	0.824	0.000	0.391	0.518
		CTRL	vs	PCL + TNF-α	1.000	1.928	0.000	0.485	0.080
		CTRL	vs	RSV-PCL + TNF-α	1.000	1.083	0.000	0.249	0.623
		CTRL + TNF-α	vs	RSV + TNF-α	1.717	0.824	0.549	0.391	0.029
		CTRL + TNF-α	vs	PCL + TNF-α	1.717	1.928	0.549	0.485	0.165
		CTRL + TNF-α	vs	RSV-PCL + TNF-α	1.717	1.083	0.549	0.249	0.069
		RSV + TNF-α	vs	PCL + TNF-α	0.824	1.928	0.391	0.485	0.031
		RSV + TNF-α	vs	RSV-PCL + TNF-α	0.824	1.083	0.391	0.249	0.123
		PCL + TNF-α	vs	RSV-PCL + TNF-α	1.928	1.083	0.485	0.249	0.036
ADAMTS4	Day 1	CTRL	vs	CTRL + TNF-α	1.000	1.635	0.000	0.220	0.037
		CTRL	vs	RSV + TNF-α	1.000	1.444	0.000	0.277	0.245
		CTRL	vs	PCL + TNF-α	1.000	1.386	0.000	0.393	0.246
		CTRL	vs	RSV-PCL + TNF-α	1.000	1.642	0.000	0.572	0.125

		CTRL + TNF-α	vs	RSV + TNF-α	1.635	1.444	0.220	0.277	0.238
		CTRL + TNF-α	vs	PCL + TNF-α	1.635	1.386	0.220	0.393	0.125
		CTRL + TNF-α	vs	RSV-PCL + TNF-α	1.635	1.642	0.220	0.572	0.756
		RSV + TNF-α	vs	PCL + TNF-α	1.444	1.386	0.277	0.393	0.760
		RSV + TNF-α	vs	RSV-PCL + TNF-α	1.444	1.642	0.277	0.572	0.369
		PCL + TNF-α	vs	RSV-PCL + TNF-α	1.386	1.642	0.393	0.572	0.383
	Day 3	CTRL	vs	CTRL + TNF-α	1.000	2.194	0.000	0.597	<0.001
		CTRL	vs	RSV + TNF-α	1.000	1.662	0.000	0.439	0.255
		CTRL	vs	PCL + TNF-α	1.000	2.570	0.000	0.668	0.246
		CTRL	vs	RSV-PCL + TNF-α	1.000	2.151	0.000	0.212	<0.001
		CTRL + TNF-α	vs	RSV + TNF-α	2.194	1.662	0.597	0.439	0.122
		CTRL + TNF-α	vs	PCL + TNF-α	2.194	2.570	0.597	0.668	0.376
		CTRL + TNF-α	vs	RSV-PCL + TNF-α	2.194	2.151	0.597	0.212	0.747
		RSV + TNF-α	vs	PCL + TNF-α	1.662	2.570	0.439	0.668	0.120
		RSV + TNF-α	vs	RSV-PCL + TNF-α	1.662	2.151	0.439	0.212	0.122
		PCL + TNF-α	vs	RSV-PCL + TNF-α	2.570	2.151	0.668	0.212	0.254
	Day 7	CTRL	vs	CTRL + TNF-α	1.000	1.265	0.000	0.485	0.376
		CTRL	vs	RSV + TNF-α	1.000	1.673	0.000	0.174	0.021
		CTRL	vs	PCL + TNF-α	1.000	1.887	0.000	1.012	0.253
		CTRL	vs	RSV-PCL + TNF-α	1.000	1.522	0.000	0.428	0.239
		CTRL + TNF-α	vs	RSV + TNF-α	1.265	1.673	0.485	0.174	0.243
		CTRL + TNF-α	vs	PCL + TNF-α	1.265	1.887	0.485	1.012	0.254
		CTRL + TNF-α	vs	RSV-PCL + TNF-α	1.265	1.522	0.485	0.428	0.380
		RSV + TNF-α	vs	PCL + TNF-α	1.673	1.887	0.174	1.012	0.509
		RSV + TNF-α	vs	RSV-PCL + TNF-α	1.673	1.522	0.174	0.428	0.380
		PCL + TNF-α	vs	RSV-PCL + TNF-α	1.887	1.522	1.012	0.428	0.382
